# Improving Real-Life Estimates of Emotion Based on Heart Rate: A Perspective on Taking Metabolic Heart Rate Into Account

**DOI:** 10.3389/fnhum.2018.00284

**Published:** 2018-07-16

**Authors:** Anne-Marie Brouwer, Elsbeth van Dam, Jan B. F. van Erp, Derek P. Spangler, Justin R. Brooks

**Affiliations:** ^1^Department of Perceptual & Cognitive Systems, Netherlands Organisation for Applied Scientific Research (TNO), Soesterberg, Netherlands; ^2^Noldus Information Technology BV, Wageningen, Netherlands; ^3^Human Media Interaction, The University of Twente, Enschede, Netherlands; ^4^Human Research and Engineering Directorate, US Army Research Laboratory, Adelphi, MD, United States

**Keywords:** heart rate, additional heart rate, non-metabolic heart rate, accelerometry, affective computing, neuroergonomics, mental state monitoring

## Abstract

Extracting information about emotion from heart rate in real life is challenged by the concurrent effect of physical activity on heart rate caused by metabolic need. “Non-metabolic heart rate,” which refers to the heart rate that is caused by factors other than physical activity, may be a more sensitive and more universally applicable correlate of emotion than heart rate itself. The aim of the present article is to explore the evidence that non-metabolic heart rate, as it has been determined up until now, indeed reflects emotion. We focus on methods using accelerometry since these sensors are readily available in devices suitable for daily life usage. The evidence that non-metabolic heart rate as determined by existing methods reflect emotion is limited. Alternative possible routes are explored. We conclude that for real-life cases, estimating the type and intensity of activities based on accelerometry (and other information), and in turn use those to determine the non-metabolic heart rate for emotion is most promising.

## Introduction: Estimating Emotional State in Real Life Using Heart Rate

Monitoring cognitive and emotional state on the basis of measures that are continuously available and do not require an individual to actively provide explicit, conscious responses would be valuable in a range of scientific and applied settings (Parasuraman and Wilson, 2008; Fairclough, 2009; Wilhelm and Grossman, 2010). The relation between physiological measures and cognitive and emotional state is complex and an active area of investigation. Factors that complicate research include the entanglement of cognitive and emotional processes, the fact that psychological concepts are not expected to map exactly on distinct physiological processes, and the difficulty to determine “ground truth” mental states (Kreibig, 2010; Brouwer et al., 2015b). On the other hand, it is without a doubt that mental state does affect physiological measures such as skin conductance, pupil dilation, heart rate variability (e.g., Brouwer et al., 2014) and the variable that we focus on here—heart rate. For instance, in our own studies we found that heart rate strongly increased in individuals who were sitting motionless in a chair after they received a message that they have to sing a song (Brouwer and Hogervorst, 2014); that heart rate was higher for individuals awaiting eye laser surgery compared to controls (Hogervorst et al., 2013) and that heart rate was reliably lower when individuals were reading emotional rather than non-emotional sections in a novel (Brouwer et al., 2015a). In these studies, we aimed to vary the intensity of the emotion (arousal—Russell, 1979), but as an illustration of the mentioned complication of entanglement of processes, the decreasing rather than increasing heart rate when reading emotional sections might have been caused by concurrent effects of attention, associated with heart rate decreases (Graham and Clifton, 1966). Still, in all of these studies we can be sure that mental processes caused the difference in heart rate, since individuals sat still under surveillance of an experimenter, thus keeping metabolic heart rate at a constant level. However, for a range of scientific and applied questions related to understanding mental processes, it is important to estimate cognitive or emotional state in daily life settings over which we have little or no experimental control (Wilhelm and Grossman, 2010) and heart rate may be affected by both metabolic and non-metabolic processes. For the remainder of this article, we will refer to “emotion” rather than “mental processes” or “cognitive and emotional state” because this is focus of most of the discussed work. We acknowledge that we (and others) cannot always clearly distinguish between these constructs.

A number of complications arise when using heart rate as an indicator of emotion in real life. These include technical and validation issues (Wilhelm and Grossman, 2010; Brouwer et al., 2015b), but also the fact that heart rate varies with a range of processes apart from emotion. Examples are food intake (Fagan et al., 1986) and circadian effects (van Eekelen et al., 2004), but most of the variation in heart rate in daily life is due to the muscles’ energy demand caused by physical activity (Grossman et al., 2004). This is not to say that these processes are unrelated to emotion; in fact, physical activity can be considered as a valuable source of information about emotion in itself. However, physical activity and emotion are not related one-on-one whereas physical activity is always strongly related with heart rate, regardless of whether the physical activity is associated with emotion.

One approach to deal with the effects of movement is to discard data associated with strong physical movements (e.g., Healey et al., 2010; Plarre et al., 2011), or focus on part of daily life that is associated with little physical movement (e.g., working at the computer as in Brouwer et al., 2017). However, this would result in a strong reduction of the range of daily life situations that can be examined. Blix et al. (1974) proposed already in 1974 to subtract part of the heart rate caused by physical activity (i.e., energy consumption of the muscles) from the overall heart rate, such that the remaining heart rate, referred to as “additional heart rate” or “non-metabolic heart rate” (Myrtek et al., 2000), would be more strongly associated with mental processes such as emotion than plain heart rate. For this to be possible, it would be required that changes in heart rate caused by physical activity and emotional activity add linearly, for which evidence has been found (Blix et al., 1974; Myrtek and Spital, 1986; Turner et al., 1988; Roth et al., 1990).

**Figure 1:**
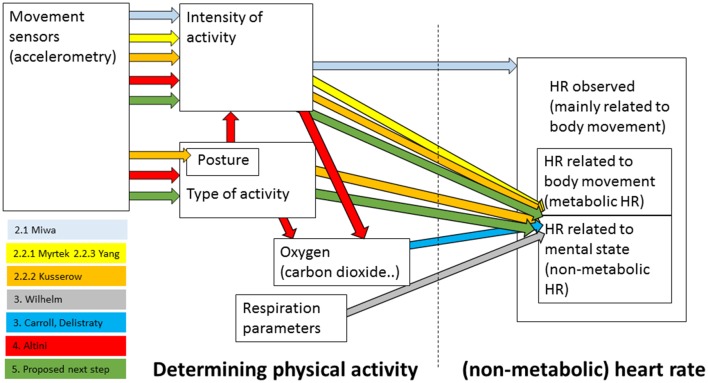
Overview of how the most important studies discussed in this article are embedded among the different variables used to estimate (non-metabolic) heart rate. Numbers preceding the names of the studies’ first authors refer to the sections in which they are discussed. In addition, the proposed next step is depicted.

Non-metabolic heart rate, or the part of the heart rate that is not purely caused by physical activity, can in principle be determined in a number of ways—starting with the choice of the sensor and variables that are used to characterize physical activity up to ways of associating this activity with heart rate and controlling for it. The aim of the present article is to explore the evidence that non-metabolic heart rate indeed reflects emotion and how non-metabolic heart rate can be best determined. The most straightforward way that is easy to apply in real life seems to be to estimate an expected metabolic heart rate on the basis of the amount of movement as given by easy-to-wear, simple accelerometers, and relate this to the observed heart rate. In “Accelerometry and Heart Rate” section we discuss evidence on the relation between accelerometry and heart rate, and specifically, in the context of research on non-metabolic heart rate. We conclude that the current methods do not provide evidence that this general method works to properly separate non-metabolic heart rate from the heart rate in general. In “Respiration and Heart Rate” section, we discuss that oxygen or respiration variables lead to a better prediction of metabolic heart rate compared to accelerometers, and thus to a more robust non-metabolic heart rate. “Accelerometry and Oxygen” section discusses the relation between accelerometry where this is used to identify types of activity and energy expenditure as recorded through analyzing breath. Work in this area seems promising and its principles may be applied to achieve a better way to determine non-metabolic heart rate on the basis of accelerometers (“Conclusions and Points for Future Work” section). “Conclusions and Points for Future Work” section also discusses an alternative approach of determining non-metabolic heart rate that may be useful in lab settings, and general points in relation to validating non-metabolic heart rate. Figure 1 gives an overview of how the most important studies discussed in this article are embedded among the different sensors and variables used to estimate (non-metabolic) heart rate. It also indicates the position of the proposed next step.

## Accelerometry and Heart Rate

### Accelerometry and Heart Rate: General Relation as Investigated Using ODBA

**Figure 2:**
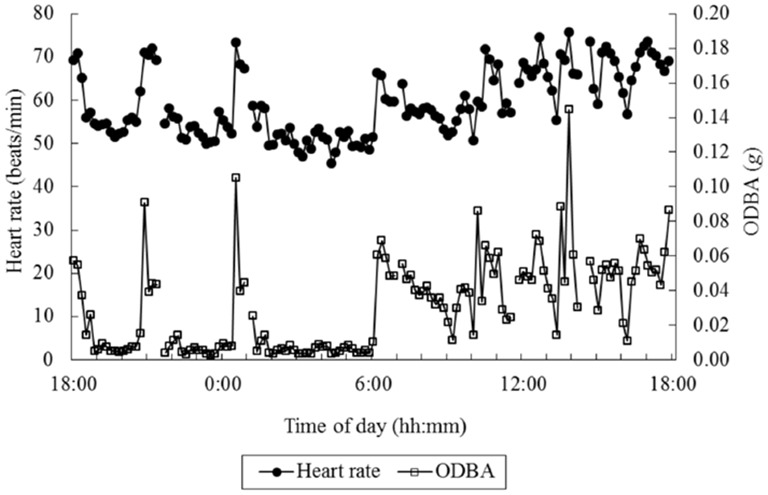
Example data of overall dynamic body acceleration (ODBA) and heart rate over 24 h in an animal. Interruptions in the data are caused by interruptions in the heart rate recording. (Figure 3 in Miwa et al., 2015; reprinted with permission).

Out of an interest in measuring energy consumption, Wilson et al. (2006) proposed the “Overall Dynamic Body Acceleration” (ODBA) as a measure reflecting energy consumption. ODBA is the summed absolute value of the output acceleration of an accelerometer from the three orthogonal axes (|ax|+|ay|+|az|). Qasem et al. (2012) argue that the vector of the dynamic body acceleration (VeDBA; sqrt(ax^2^+ ay^2^+ az^2^)) may better reflect energy consumption. While most ODBA and VeDBA studies relate these measures to energy consumption as estimated by oxygen consumption (see also next sections), Miwa et al. (2015) used heart rate as a proxy for energy consumption. They investigated the relation between ODBA and VeDBA on the one hand and heart rate on the other hand. Grazing animals (cows, goats and sheep) were fitted with a heart-rate sensor as well as with an accelerometer on the back, at around shoulder height. Figure 2 shows an example of simultaneously recorded ODBA and heart rate over 24 h in an animal. ODBA correlated with heart rate better than that the number of steps correlated to heart rate, and the same or slightly better than that VeDBA correlated with heart rate. The R-squared of a Generalized Linear Model on the complete set of data, including random effects of species and individual animals, reached 0.87. This suggests that for non-human animals, arguably without much variation in types of activity, simple accelerometry measures can predict heart rate quite well, which is hopeful for the possibility to estimate and subtract metabolic heart rate.

### Accelerometry for Determining Non-metabolic Heart Rate

The literature of subtracting out heart rate caused by physical activity using accelerometry to arrive at a heart rate measure that better reflects emotion has been dominated by the Additional Heart Rate algorithm developed by Myrtek and colleagues. As observed by Kusserow et al. (2013), who built upon this algorithm, no comparable arousal estimation algorithm exists that can be applied in natural daily living. Recently, Yang et al. (2017) took a slightly different approach relating accelerometer data to non-metabolic heart rate. The three approaches are described below.

#### Myrtek: Most-Used Algorithm of Determining Non-metabolic Heart Rate

Myrtek et al. (1988) proposed an algorithm for determining non-metabolic heart rate using accelerometers (usually one located at the chest and one at the thigh) to probe physical activity. Myrtek and colleagues published a large body of research that is mostly concerned with online identification of moments at which increases in heart rate are due to emotion rather than (only) to movement.

Non-metabolic heart rate is computed as follows, where the explanation is largely taken from Streb et al. (2015) who indicated working with non-metabolic heart rate as defined by Myrtek (2004):

1. The actual heart rate of minute i (HRi) is compared to the average of the previous 3 min (HRPR) resulting in the actual change in heart rate at minute i (HRCi).
2. Next, physical activity at minute i (“ACTi”) is factored in. From the original publications by Myrtek and colleagues it is unclear how ACTi is determined exactly. ACTi was quantified by Streb et al. (2015) as vectorial additions of the two sensors. Kusserow et al. (2013) describe ACTi as vectorial addition (where the sensor at the thigh is only used for the saggital direction) followed by additional (log) transformations.
3. If there is no substantial increase in physical activity (< = 10 units where ACTi is compared to the average ACT of the preceding 3 min) an emotional event is postulated if HRCi = >3.
4. If there is a substantial increase in physical activity (> = 10 units), the minimal rise in heart rate used to indicate an emotional event increases as well. Rather than exceeding 3, the HRCi then needs to exceed HRPR+HRPLUSi where HRPLUSi = (ACTi+90)/PAR. Parameter PAR affects the number of signaled emotional events and can be adapted online such that in online studies where participants are asked for a response at a supposedly emotional event, all participants receive an about equal number of alerts (e.g., Myrtek and Brügner, 1996). It is also often set (in offline analysis) at a fixed value of 30.
5. The non-metabolic (additional) heart rate AHRi is determined by the actual increase in heart divided by the heart rate increase that is minimally required to signal an emotional event: AHRi = HRCi/HRPLUSi.
6. but only if HRCi > = HRPLUSi., i.e., the algorithm only considers deviations of HR due to physical activity that are positive.

Note that the way information about body movement is extracted from accelerometers in this algorithm is similar to the computation of ODBA and VeDBA, except that here two accelerometers are used, and a subsequent log and linear transformation may be included. Myrtek (1990) and Myrtek et al. (1995) report correlations of between 0.70 and 0.73 between heart rate and physical activity; Myrtek et al. (2005) mention correlation coefficients up to 0.81, indicating that (also with effects of emotion included) there is quite a strong relation between body movement as determined by the algorithm and heart rate. Note that the step from heart rate explained by physical activity to non-metabolic heart rate is not a simple subtraction but includes some constants or parameters that seem to have been chosen to detect a “reasonable” number of emotional events.

#### Kusserow: Extending Myrtek’s Algorithm by Taking Into Account Changes in Posture

Kusserow et al. (2013) observe that a systematic evaluation and optimization of the functional elements and parameters of the algorithm by Myrtek and colleagues has not been performed. They argue that the current Myrtek algorithm does not take into account changes in heart rate due to (changes in) postures, such that changes in posture are incorrectly labeled as “emotional arousal.” They thus elaborated the model by including different primitives related to posture (sit, stand, walk, bend front, lean back, upright) derived from the chest and thigh accelerometer data using machine learning. Kusserow et al. (2013) algorithm results in less emotional arousal events when it is “activity aware” (i.e., taking into account posture) compared to when it is not, especially for situations with a high level of physical activity. They also extended the algorithm such as to obtain measures of duration.

#### Yang: An Alternative Algorithm

Yang et al. (2017) propose a model to predict heart rate based on an accelerometer data from a sensor on the chest. Their algorithm is based on a formula predicting heart rate demand on the basis of an individual’s minimum heart rate (HRmin) and maximum heart rate (HRmax), as well as exercise intensity (Iintensity; Karvonen et al., 1957):

HRdemand = (HRmax − HRmin) * Iintensity + HRmin

In their study, HRmax has been fixed per age (She et al., 2015) and the HRmin is as recorded in rest. From HRmin and gender, a parameter lamda is determined (Zakynthinaki, 2015) reflecting overall cardiovascular condition.

Like the other algorithms, the exercise intensity is based on the acceleration vector sqrt(ax^2^+ ay^2^+ az^2^). This is subsequently divided by the maximum achievable velocity of the individual, which is determined by lamda and a fixed constant (Zakynthinaki, 2015). In determining the exercise intensity, Yang et al. (2017) also model the effect of blood lactate (that accumulates during exercise and also causes an increase in heart rate: Zakynthinaki, 2015) where again they use physical movement to do this. Finally, they model the speed at which the heart adapts taking into account lamda and a fixed constant.

Non-metabolic HR is determined by subtracting the observed heart rate from the predicted one. Yang et al. (2017) further translate this into a “mental state arousal level” by mapping the non-metabolic heart rate on [−1, 1] using the minimum and maximum heart rate.

Yang et al. (2017) present example graphs of predicted and observed heart rate during several activities (lying, sitting, walking, ironing, vacuum cleaning, Nordic walking, running, rope jumping and cycling). As far as the overall prediction of heart rate is concerned, they conclude that the model works well for some activities, but not when the chest accelerometer does not capture the movement optimally (such as in cycling and vacuum cleaning) and not after vigorous activities such as Nordic walking.

### Evidence That Accelerometry Based Non-metabolic Heart Rate Reflects Emotion

Several studies take it as a given that non-metabolic heart rate determined in ways as described above reflects emotion and build further conclusions on it. For instance, Wilhelm and Grossman (2010) motivate the importance of ambulatory monitoring by referring to figures that show large heart rate increases compared to physical activity as recorded by accelerometry in real-life situations—watching a soccer game and giving a speech. Another example is a study by Streb et al. (2015). They recorded non-metabolic heart rate following the Myrtek algorithm in children attending different types of educational environments. Streb et al. (2015) found higher non-metabolic heart rate in environments that emphasized social relatedness and autonomy, which led them to certain educational recommendations. However, before such conclusions can be drawn we need to know whether or under what circumstances we can assume that the various types of determining non-metabolic heart rate reflect emotion. If this is not clear, computed non-metabolic heart rate could be attributed to emotion while actually being caused by, for example, confounding differences in speech or movements that are not captured well enough by the accelerometers. See Kusserow et al. (2013) who showed incorrect labels of emotion due to posture change that was not captured, and Linden (1987) reporting that unemotional speech (which will not affect accelerometry) can increase HR with 5–10 bpm. For the Streb et al. (2015) study, it is likely that children in the environments emphasizing social relatedness and autonomy talked and moved (changed posture) more, and the examples given by Wilhelm and Grossman may have suffered from the same problem.

Below, we review the evidence of studies that explicitly aimed to test whether non-metabolic heart rate as determined in a certain way using accelerometry is indeed associated with emotion.

Myrtek and colleagues prompted participants during daily life activities to rate excitement and enjoyment on 4- or 5-step rating scales (three studies described in Myrtek and Brügner, 1996) and in addition, the type of emotion (no emotion, disgust, anger, happiness, anxiety, sadness, surprise; Myrtek et al., 2005). The times they were prompted were either times that were identified as emotional through non-metabolic heart rate or times that this was not the case. Numbers of participants in these studies varied between 49 and 323; prompts were given every 10–20 min (except during sleep). Recording duration was usually 23 h. Myrtek and colleagues did not find differences in the frequency and quality of reported emotions depending on whether the probe was elicited by non-metabolic heart rate or not. Also, Myrtek et al. (2005) did not find that some individuals were more accurate in indicating emotions than others; participants mainly differed as to the overall degree of feeling emotional. Thus, this approach has not provided support for non-metabolic heart rate as determined by the algorithm proposed by Myrtek (2004) as an indicator of emotion.

However, the difficulty with comparing physiological correlates of emotion with self-reported emotion is that it is not* a priori* clear, which is closer to the “true” emotion. One of the frequently mentioned motivations of using physiology as an indicator of emotion is the unreliability of self-report. In this line, Myrtek and Brügner (1996) and Myrtek et al. (2005) argue that participants’ emotional self-report is affected by subjective hypotheses and schemata that are usually clear-cut in laboratory settings, therewith enhancing correlations between self-reported emotion and physiology, but not so in daily life (Pennebaker, 1982; Rimé et al., 1990).

Another approach is to compare non-metabolic heart rate between different groups of participants or different conditions where* a priori* different intensities of emotions are expected. Myrtek et al. (2005) mention a range of such studies. However, it is not clear that non-metabolic heart rate shows a clearer difference than plain heart rate; rather the opposite seems to be the case. For instance, Myrtek and Brügner (1996) show that non-metabolic heart rate is higher when watching erotic movies compared to comedy. This is the case for little physical activity (sitting) and strong physical activity (cycling). However, the effect of the type of movie was, or at least tended to be, stronger in both sitting and cycling conditions for plain heart rate compared to non-metabolic heart rate (where in the sitting condition the difference in movement went in the direction of less activity when watching the erotic movie compared to the comedy).

Kusserow et al. (2013) related estimated emotional arousal phases to 12 annotated daily activities (such as eating, conversing, housework, office desk) to examine whether estimated arousal results seemed plausible, but as acknowledged by the authors, it is hard to judge whether this was the case. They did not find congruence between estimated arousal and subjectively perceived arousal, as indicated by four participants using questionnaires that they had filled out at random, self-chosen times (with on average about 2–3 h between them; 32–64 h of recording per participant). Kusserow et al. (2013) indicate that evaluating their algorithm as to whether it correctly indicates emotion was not the main purpose of their study, and their first results did not provide evidence in this respect.

**Figure 3:**
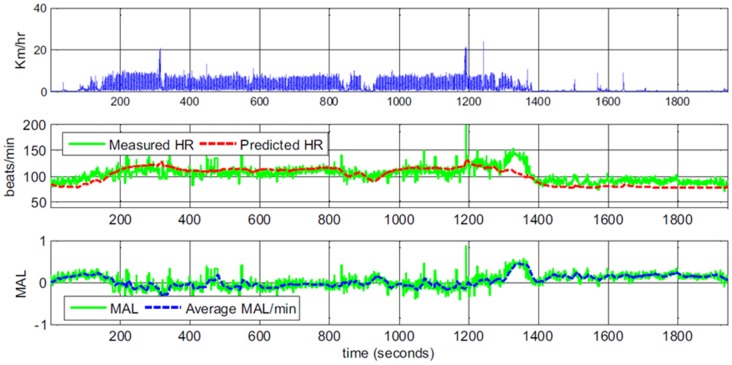
Example data of body movement, heart rate and derived mental arousal level (MAL) of a participant walking home from work. The data was interpreted as the participant being a little excited to leave work in the beginning and being irritated at second 1250 because of a phone call. The continued positive MAL after the participant arrived at home and sat on the sofa (second 1360) was interpreted as the participant thinking about the phone call (Figures 1, 4 in Yang et al., 2017; reprinted with permission).

Yang et al. (2017) show example graphs plotting predicted and observed heart rate both for participants performing different activities in the lab, as well as for a participant in real life. Thirty-minute “snapshot” example graphs suggest a rather good fit between predicted and observed heart rate. At times when predicted heart rate seems lower than observed, the authors provide explanations such as the participant is walking part of a route that she knows particularly well; when the predicted heart rate seems higher, the suggestion is that this is due to arousal caused by chatting with a friend or a stressful phone call (a reprinted example graph is shown in Figure 3). However, there is no independent measure of emotional arousal.

In sum, we are not aware of a study that indicated evidence of non-metabolic heart rate estimated using accelerometry to be associated with emotion. It may not be possible to capture enough of the necessary information using (a limited number of) accelerometers, or the algorithms used up until now to extract the information may not be suitable. The algorithms by Kusserow et al. (2013) and Yang et al. (2017) have not been tested extensively.

## Respiration and Heart Rate

While accelerometry may potentially be good enough to predict heart rate caused by physical activity, some obvious limitations already preclude a straightforward relation under all circumstances. The capturing of movement depends on the placement of the sensors—as mentioned before when discussing the study by Yang et al. (2017), an accelerometer on the trunk will suggest little exercise when working out on a home trainer (see also Crouter et al., 2006). Isometric exercise cannot be captured by body movements—lifting a heavy box or a light box would be similar in terms of accelerometry, but not in metabolic demand. Metabolic demand as estimated using oxygen consumption does not suffer from these disadvantages (Wilhelm and Grossman, 2010). In fact, and as previously discussed, non-metabolic heart rate was originally determined by separating out heart rate due to muscular movement as estimated using oxygen consumption (Blix et al., 1974). Using oxygen consumption as a proxy of physical movement for determining non-metabolic heart rate would only work if emotion does not affect oxygen uptake in a similar way than it affects heart rate (or if oxygen uptake and heart rate are not very tightly connected). This has been established (Carroll et al., 1986; Delistraty et al., 1991). These studies related oxygen uptake to heart rate under neutral conditions with varying physical activity, and during a stressful cognitive task with varying physical activity. Heart rate in the stressful condition was higher than predicted from oxygen uptake. However, oxygen consumption cannot easily be measured during daily life. As an alternative, Wilhelm and Roth (1998a,b) used respiration bands at the upper thorax and the abdomen to estimate the part of heart rate caused by movement. They refer to Delistraty et al. (1991) who showed a correlation close to 1 between oxygen consumption and minute ventilation at low and moderate levels of aerobic exercise (up to 60% of maximal work capacity). Bands were individually calibrated using a spirometer and the association between physical activity and respiration was individually determined by regression analysis on ventilation and heart rate data originating from cycling at different intensities. A mean R-squared of around 0.80 between observed and predicted heart rate during the cycling calibration procedure was found, which is much higher than the previously mentioned correlation when accelerometry was used in humans. Non-metabolic heart rate was determined by subtracting the heart rate as predicted by respiration data from the observed heart rate.

**Figure 4:**
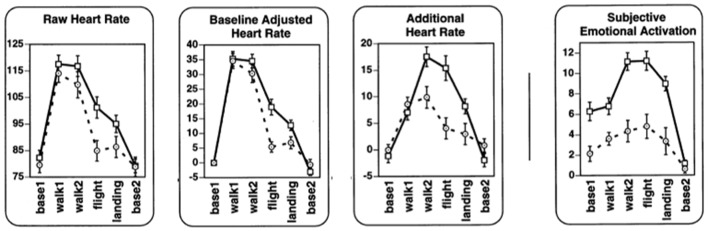
Raw heart rate, baselined heart rate, additional (non-metabolic) heart rate and subjective emotion during respectively pre-flight baseline as recorded in a hospital; walking from the hospital to the car; walking from the terminal to the airplane; flight; landing; post-flight baseline. Data from flight phobic participants are represented by squares and a solid line; data from control participants are represented by circles and dashed lines (Modified Figure 2 in Wilhelm and Roth, 1998b; reprinted with permission).

Wilhelm and Roth (1998a,b) validated whether non-metabolic heart rate thus determined reflected emotion by recording from flight phobics and controls while they were walking, entering a plane, flying and landing (Figure 4). Compared to raw heart rate and baselined heart rate, non-metabolic (“additional”) heart rate was higher for phobics than for controls in most phases. Also, other statistical tests indicated that non-metabolic heart rate was more sensitive to distinguish the more emotional (phobic) group from the control group compared to the other heart rate measures. Rated subjective anxiety and excitement (“subjective emotional activation”) were shown to be associated with non-metabolic heart rate. The advantage of non-metabolic heart rate over plain heart rate is most clear when recorded during movement (e.g., walking to the airplane) since obviously, during these periods, there was something to correct for.

These studies (arguably covering a quite extreme case of comparing flight phobics to controls during an actual flight scenario) genuinely show that non-metabolic heart rate reflects emotion more accurately than plain heart rate, where the non-metabolic heart rate was determined using respiration bands to determine heart rate as affected by metabolic demand.

## Accelerometry and Oxygen

While non-metabolic heart rate has thus been shown to be informative about emotional state outside the lab, this was shown using respiration parameters and not using accelerometry. In the literature on relating accelerometry to energy expenditure (including ODBA work), numerous models have been developed (Corder et al., 2007), usually based on linear regression analysis. While these models work to some extent, they suffer from the same limitation that the relation between accelerometry and heart rate suffers from, and which may be the very reason why non-metabolic heart rate based on simple accelerometry was not shown to be successful: body movement does not map directly onto metabolic need. Bonomi et al. (2009) proposed to harness accelerometry as a predictor of energy expenditure in a fundamentally different way, reminiscent by what Kusserow et al. (2013) did for heart rate. They proposed to use accelerometry for activity recognition and combine this with known data on the energy cost of these activities (metabolic equivalent table (MET) compendium values: Ainsworth et al., 2000).

**Figure 5:**
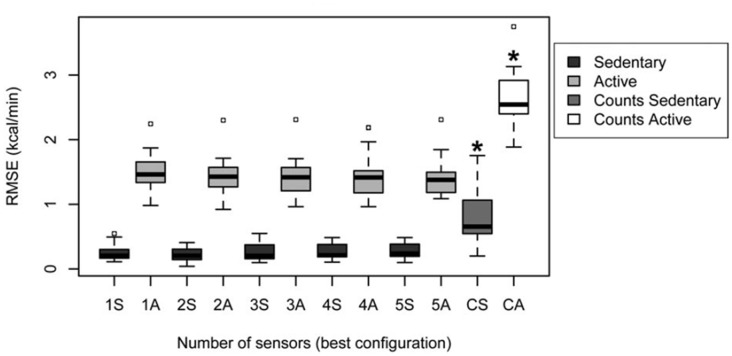
Root-mean-square-error (RMSE) of energy expenditure estimation following the traditional “counts based” method for sedentary and active clusters (the two data distributions on the right, marked CS and CA); and the “activity specific” method as a function of the number of used accelerometers, for both sedentary (dark gray) and active (light gray) behavior (Figure 4B in Altini et al., 2015; reprinted with permission).

Altini et al. (2015) give an overview of methods that have been followed to relate accelerometry to energy expenditure, indicating that activity-specific methods outperform the traditional method. In their study, they directly compare energy expenditure estimation performance between different methods and different accelerometer positions. They had participants perform a large number of varying activities at different intensities (such as lying down resting, desk work, cooking, washing windows, running) that were clustered into “sedentary” and “active” behavior, where these two clusters were again subdivided into respectively lying, sitting and standing; and high whole-body motion, walking, biking and running. Participants wore five accelerometers at different body locations, and a calorimeter (a wearable device covering nose and mouth to measure breaths) to measure energy expenditure. In what they refer to as the “counts-based method,” accelerometer features (0.1–10 Hz band passed values summed over the three axes) are regressed to energy expenditure values without knowledge of activity type (i.e., the traditional method, similar to the methods relating accelerometry to heart rate described in Section Introduction: “Estimating Emotional State in Real Life Using Heart Rate”). For activity-specific estimation methods, the type of activity is estimated using a range of accelerometry features and support vector machine classification. Then either a MET was used to look up the corresponding energy expenditure, or activity-specific linear models relating accelerometry to energy expenditure are used. In the latter version, the accelerometry features used depend on the type of activity. All methods also include anthropometric characteristics (body weight and resting metabolic rate). Models were evaluated using leave-one-participant-out cross validation. Altini et al. (2015) show that energy expenditure can be estimated best by activity-specific estimation methods (Figure 5), where using accelerometry based models depending on the activity outperformed MET only for the active cluster (containing activities that can be performed at different intensities which is hard to capture in a look-up table). They conclude that two accelerometers at proper locations (chest and wrist) suffice for activity recognition, one sensor (chest) is enough for to estimate subsequent activity dependent energy expenditure for active activities.

## Conclusions and Points for Future Work

In this survey, we explored to what extent non-metabolic heart rate has been shown to reflect emotion, and how non-metabolic heart rate can be best determined. The existing literature suggests that relating movement intensity of accelerometers “directly” to metabolic heart rate is too course a method in uncontrolled movement circumstances to consecutively obtain non-metabolic heart rate estimates that are informative of emotion. Studies using respiration rather than accelerometry (where more or less wearable bands can be used) to estimate metabolic heart rate did show that non-metabolic heart rate relates to emotion. Still, from a practical viewpoint, respiration bands are not desired. As highlighted through studies relating activity type to energy expenditure (Altini et al., 2015), a promising approach would be to classify actions using accelerometers or other wearable sensors (Wong et al., 2007; Cornacchia et al., 2017) and predict heart rate due to physical activity from there. A side advantage of performing activity recognition rather than using respiration-related variables to determine non-metabolic heart rate is that, as also mentioned in the introduction, activity type and duration are often variables of interest in studies on emotion by themselves. Given the fact that speech affects heart rate apart from emotion but cannot be captured with movement sensors, an acoustic sensor may be valuable for activity recognition for the purpose of determining non-metabolic heart rate.

Another straightforward approach that does not depend on correct modeling of activity recognition, or on correct modeling of the relation between variables indicative of metabolic need and heart rate caused by physical activity, is to create an own look-up table by having individuals perform the body movements of interest under circumstances that are known to be low in emotion. The individual’s heart rate recorded at that time can be subtracted from the heart rate recorded during the same activity as determined by the design of an experiment in a (possibly) emotional setting (Brouwer et al., 2018). Obviously, this requires extra data collection and cannot be generalized to all daily life circumstances. However, it may be a tool in naturalistic experiments examining emotions in a continuous, implicit way.

The current manuscript focuses on non-metabolic heart rate. The variation of time between subsequent heart beats, or heart rate variability, has been associated with psychological (and physical) stress where a decrease in heart rate variability correlates with an increased level of stress (McEwen, 2001; Brouwer et al., 2014; Javorka et al., 2018). If the quality of the recording of heart rate allows, heart rate variability may be used as an additional informative variable about an individual’s mental state. Heart rate variability is affected by physical movement (and associated changes in respiration) as well, but the effects of movement may be taken into account in similar ways as described. First studies on non-metabolic heart rate variability have been performed by Verkuil et al. (2016) and Brown et al. (2017).

While we think that the global method as proposed will lead to a better determination of non-metabolic heart rate than the methods used so far, the modeling will still not be exact. For instance, current heart rate will be affected to varying degrees by recent physical activity where such effects will be stronger or longer lasting when this activity was intense. It is important to evaluate any new models on non-metabolic heart rate in different contexts, or at least, in the context of interest. For this, independent measures of emotion are required such as subjective ratings or conditions or groups that can* a priori* be expected to differ with respect to experienced emotion. While obtaining ground truth emotion is challenging (André, 2011; Brouwer et al., 2015b), such measures should be used to provide evidence for the potency of the method to estimate emotion, and to compare different methods of determining non-metabolic heart rate.

Knowing an individual’s emotion continuously can be desired in a wide range of cases. However, application of any measure reflecting this should be viewed in the light of its accuracy, its cost-benefit trade-off compared to alternative measures, as well as ethical considerations (Brouwer et al., 2015b). For continuous non-metabolic heart rate, applications could be in obtaining a continuous characterization of stress or affective intensity in well-defined tasks that involve light to moderate movements (e.g., complex tasks in air traffic control, or in cooking a dish- Brouwer et al., 2018). This can support the design of tasks, interfaces and products. A real-time determination of non-metabolic heart rate may be used in real-time interventions by virtual coaches, e.g., to support individuals with an anxiety disorder or addiction.

## Author Contributions

A-MB conceived the idea of the manuscript and wrote the first draft of the manuscript. All authors contributed to manuscript revision, read and approved the submitted version.

## Conflict of Interest Statement

The authors declare that the research was conducted in the absence of any commercial or financial relationships that could be construed as a potential conflict of interest.
